# Clinical and morphological characteristics of head-facial haemangiomas

**DOI:** 10.1186/1746-160X-3-12

**Published:** 2007-02-23

**Authors:** Giorgio Iannetti, Andrea Torroni, Stefano Chiummariello, Carlo Cavallotti

**Affiliations:** 1 Maxillo-Facial Surgery Unit, Medical Faculty, University "La Sapienza" Rome, Italy; 2 Plastic Surgery Unit, Medical Faculty, University "La Sapienza" Rome, Italy; 3 Department of Anatomy, Medical Faculty, University "La Sapienza" Rome, Italy

## Abstract

**Background:**

Haemangiomas of the head or face are a frequent vascular pathology, consisting in an embryonic dysplasia that involves the cranial-facial vascular network. Haemangiomas show clinical, morphological, developmental and structural changes during their course.

**Methods:**

The clinical characteristics of head-facial haemagiomas were studied in 28 individuals (9 males and 19 females) admitted in our Hospital. Sixteen of these patients(n = 16) underwent surgery for the removal of the haemangiomas. All the removed tissues were transferred in experimental laboratories for the staining of microanatomical details, somatic and visceral nerve fibres, adrenergic and catecholaminergic nerve fibres. Beta-adrenergic receptors were died with a fluorescent method. All results were submitted to the quantitative analysis of images and statistical evaluation of the data.

**Results:**

The morphological results revealed numerous micro-anatomical characteristics of the haemangiomatous vessels. The somatic and visceral nerve fibres were poor and located exclusively in the adventitial layer. There was a marked decrease of adrenergic nerve fibres in the haemangiomatous vessels. The fluorescence of catecholaminergic nerve fibres and the overall area of fluorescent structures were also decreased in haemangiomatous vessels. Beta adrenergic receptors are strongly decreased in haemangiomatous vessels. The morphometrical analysis of images and statistical evaluation of the data confirmed all our experimental results.

**Conclusion:**

The catecholaminergic innervation of the human haemangiomatous vessels comprises nerve fibres containing the main catecholaminergic neurotransmitters that are sympathetic in nature. These neurotransmitters are closely related to beta-adrenergic receptors.

The sympathetic nervous system plays a key role in the control of the vascular bed flow and vascular motility in both normal and haemangiomatous vessels.

## Background

A bibliographic research on med-line for the word "haemangioma " yields 1440 references in the last three years (2004–2006). The association of the term "head-facial" strongly reduces this number. The addition of the term "nervous system" further reduces the number of references to a mere seven. None of these seven references concerns the catecholaminergic innervation of haemangiomas. Haemangiomas are benign lesions of blood vessels that faithfully create well formed vascular channels. Usually present from birth, they may represent congenital hamartomas or benign neoplasms arising in a congenital defect [1]. In the newborn, they may be so small as to be in-visible. These lesions are considered by many researchers to be hamartomas that grow as the body develops [2]. However, these lesions may even arise spontaneously as true neoplasms in children or adults [3]. As the body grows, these masses become highly visible becoming several centimetres in diameter. In many instances, they cease to enlarge and enter a period of apparent dormancy [4]. Although haemangiomas are usually of little clinical relevance, when present in the brain, they are potential sources of increased intra-cranial pressure or haemorrhage. Malignant transformation of a cavernous haemangioma is an extremely rare occurrence [5]. The venous wall ultra-structure in generalized venomegaly and/or arteromegaly was studied in our laboratories from many years [6,7], including the sympathetic innervation of the mega-vessels [8]. Cunha and co-workers studied, with histochemical, immunohistochemical and ultrastructural methods, the innervation of malformative cortical vessels in Sturge-Weber disease. These authors suggested that the abnormal vessels were innervated exclusively by noradrenergic sympathetic nerve fibres [9].

Since little is known about the innervation of haemangiomas, although adrenergic receptors are known to play a role in the occurrence and healing of haemangiomas, the aim of this study was to shed light on the catecholaminergic and adrenergic nerve fibres and the beta-adrenergic receptors of the haemangiomas.

## Materials and methods

The Clinical units of Plastic Surgery, Maxillo-Facial Surgery and Neurotraumatology of our University enrolled, in the last five years, 28 patients with cranio-facial haemangiomas (n = 28). All the patients were studied for clinical or diagnostic reasons. Table 1 contains basic information of the patients such as age and sex. All the patients underwent a thorough clinical study. Every case was evaluated with ultrasound imaging, CT and MRI to obtain a complete diagnostic study. In presence of urgent, complex lesions that would need a more aggressive treatment the therapy was planned in collaboration with other Clinical Units involved in these studies. All the lesions were recorded according to photographic and objective clinical evidence. The following therapies were used:- embolism – laser therapy – other surgical techniques [10]. The laser used was an ND: YAG laser (neodymium :yttirium-aluminium-garnet) used with fluorescence of 400–600 J/cm2 with pulse duration of 0,5 sec. These settings were selected on the basis of good results in literature [11-15].

**Table 1:** Clinical data on patients enrolled in the present experiment

Patient	Sex	Age	Operated	Patient	SEX	Age	Operated
1	M	54	YES	15	M	82	NO
2	F	49	YES	16	F	77	NO
3	M	28	NO	17	M	58	YES
4	M	34	NO	18	M	50	YES
5	M	56	YES	19	M	54	YES
6	F	42	YES	20	F	72	NO
7	F	40	YES	21	F	76	NO
8	F	79	NO	22	F	54	YES
9	M	66	NO	23	M	42	YES
10	M	58	YES	24	M	40	YES
11	M	56	YES	25	M	46	YES
12	M	62	YES	26	M	48	YES
13	F	60	YES	27	F	52	YES
14	F	78	NO	28	F	54	YES

The surgical techniques varied according to lesion extension and location. Serial or simple excision was performed. If necessary the reconstruction of the ablated tissues was performed with local or microsurgical flaps. An immediate post-surgical and 3-6-12 month follow-ups was performed and documented.

16 patients were underwent conventional surgical therapy for the removal of haemangiomas and fresh samples of removed tissues were studied in our experimental laboratories. As mentioned previously, samples of the hemangiomatous vessels were obtained from haemangiomas removed in case of open surgery avoiding of course any potential risk for the patients, all procedures were in accordance with the ethical standards of the responsible Committee on human experimentation and with the Declaration of Helsinki (1964) of the World Medical Association (amended in 1988) [16]. As "normal" vessels we used small samples of vessels of the same patient, and in the same zone, considered as normal after an accurate examination.

The fresh material was fixed, dehydrated, paraffin-embedded and cut for morphological staining. The microanatomical details were displayed using the hematoxylin-eosin as dye, as usual in morphological laboratories [17]. Moreover, all somatic and visceral nerve fibers were stained using the method of Bodian [18]. This method can be used to verify that a stained structure is nervous in nature. In fact, it stains all nerve fibres and neuro-fibrils. The adventitia was separated with scissors from other layers and stretched on a slice. After fixation, the sections were treated with: 1) 1% Protargol solution (colloidal silver), 2) reducing solution (Hydroquinone + sodium sulphite), 3) 1% Gold chloride solution, 4) 2% oxalic acid solution and counterstained with 0.03% aniline blue. The nerve fibers and neuro-fibrils are stained in black.

Other fresh samples were refrigerated and preserved until use. On these samples we performed the staining of the adrenergic nerve fibres (ANF) by Falck technique, using the fluorescence induced by formaldehyde vapours [19] and/or with glyoxylic acid technique (GIF) for catecolaminergic nerve fibres [20] Moreover, on fresh samples we performed also the staining of adrenergic receptors[21]. ANF staining : Small samples can be dissected and treated to reveal adrenergic nerve fibres under formaldehyde vapours. After staining, samples can be examined by fluorescence microscopy using a Carl Zeiss photomicroscope (PMQ-II; Jena; Germany) provided with an epi-illumination system for fluorescence observations.

Samples are photographed using black and white pan F18 DIN film. The a-specific fluorescence can be barred with special filters.

A glyoxylic acid-induced fluorescence technique is used for the staining of catecholaminergic nerve fibres (CNF) (20). Briefly, immediately before the use the staining solution can be prepared by adding a solution of 0.236 M potassium phosphate (pH 7.4) 0.2 M sucrose and 1% glyoxylic acid.

This staining is named sucrose, phosphate, glyoxylic acid (SPG). The slides with samples can be immediately dipped in this solution for 5 min. To assure a comparable fluorescence it is important to standardize times and temperatures without intervals. After staining, the sections must be drained, covered with non-autofluorescent immersion oil, heated at 95°C for 5 min., observed and photographed to prevent diffusion and photodecomposition of the fluorescence. The sections can be examined and photographed under a Zeiss photomicroscope equipped with exciter and barrier filters and with a mercury lamp for observation of fluorescence.

Beta-adrenergic receptors staining: The location of beta-adrenergic receptors in the wall of angiomatous vessels is identified by fluorescent staining using a beta-blocker drug (Pindolol-Visken LB 46 Sandoz Basilea CH) conjugated with a fluorochrome (fluoresceine isothiocyanate = FITC). The biological activity of the fluorescent pindolol (FPIN) has been previously tested both "in vitro" and "in vivo", by studying its cardio-vascular activity and dosing the nor-epinephrine-dependent adenilate-cyclase. After binding, the pharmacological effect of FPIN is reduced by 30%. FPIN can be used to locate beta-adrenergic receptors on fresh-cut sections of human angiomatous vessels. The specificity of this reaction has been previously assessed pre-treating some sections with non-fluorescent pindolol and then exposing them to FPIN or by treating samples with reaction-inhibiting agents. We used propanolol (which blocks both b1 and b2 receptors) [22], ICI 118 551 (which blocks b2 receptors alone) [23] and CGP 20712A (which blocks only b1 receptors) [24]. The sections were stretched on a microscope slide and treated for 30' at room temperature with the afore-mentioned beta-blocker substance conjugated with fluoresceine isothiocyanate (FITC). Thereafter the samples were washed, for 10', in an isotonic solution of 10% polyvinylpyrrolidone (PVP), thermostat dried at 37% and mounted in a water-soluble medium. The corners of the covering slide were treated with boiling paraffin and enamelled. Observations were performed under a U.V. Zeiss photomicroscope using the following combinations of exciting and barrier filters: UG1 with 41/44 and BG12 with 53/44.

To stain the sections it is possible to use either an anti beta-blocker antibody conjugated with FITC or a beta-blocker conjugated, directly, with FITC. As the results yielded, in the first case we observed less evident results, we recommend the use of fluorescent staining with the beta-blocker conjugated with FITC.

Quantitative analysis of images (QAI). In order to evaluate the amount of staining, a quantitative analysis of the intensity of the histofluorescent staining can be performed on photographs (to avoid photodecomposition) by means of a Quantimet Analyzer (Leica). The control values from samples incubated without fluorochrome, were considered as "zero". The following parameters can be measured 1)total area of fluorescent structures, 2)number of nervous varicosities, 3)number of crossings or intersections of nerve fibres. All these values can be expressed as conventional unit (C.U.). Each photograph can be examined separately by calculating the standard error of the mean (=S.E.M.). The final values undergo statistical analysis. The values reported in our experiments represent the intensity of staining for each type of tissue and are expressed in conventional units (C.U.) ± SEM. Further details on QAI are reported in Manual of the Quantimet Leica 500 image analyser [25]

### Statistical analysis

To ascertain the significance of QAI, standard Error of Mean (SEM), probability index (p) and Student t' test were performed.

## Results

Clinical studies we conducted showed that the vascular bed of haemangioma is connected to the surrounding blood vessels through a solitary afferent and efferent channel. This interesting finding explains why spontaneous or induced thrombosis of the connecting vessels may lead to thrombosis of the whole haemangioma with consequent obliteration of the lumen of the entire lesion.

On samples coming from surgical theatre, as said above, we performed numerous experiments. The following micro-anatomical details emerged from the haematoxiline-eosin staining: the mass of the haemangiomas is sharply defined, but not encapsulated, and made up of large, cavernous, vascular space, partly or completely filled with fluid blood separated by a scant connective tissue stroma. Intra-vascular thrombosis or rupture of channels may modify its histologic appearance (Fig, 1).

**Figure 1:**
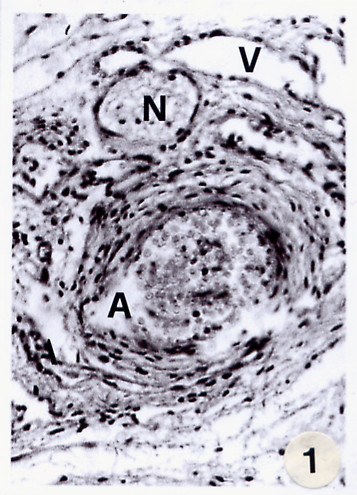
Light microscopy of haemangiomatous vessels of the face stained with haematoxyline-eosine revealing micro-anatomical details. A = artery; V = vein; N = nerve. Both the endothelial and medium layers of the artery wall are uneven discontinuous and non homogeneous (magnification 100×; bar 100 μm).

**Figure 2:**
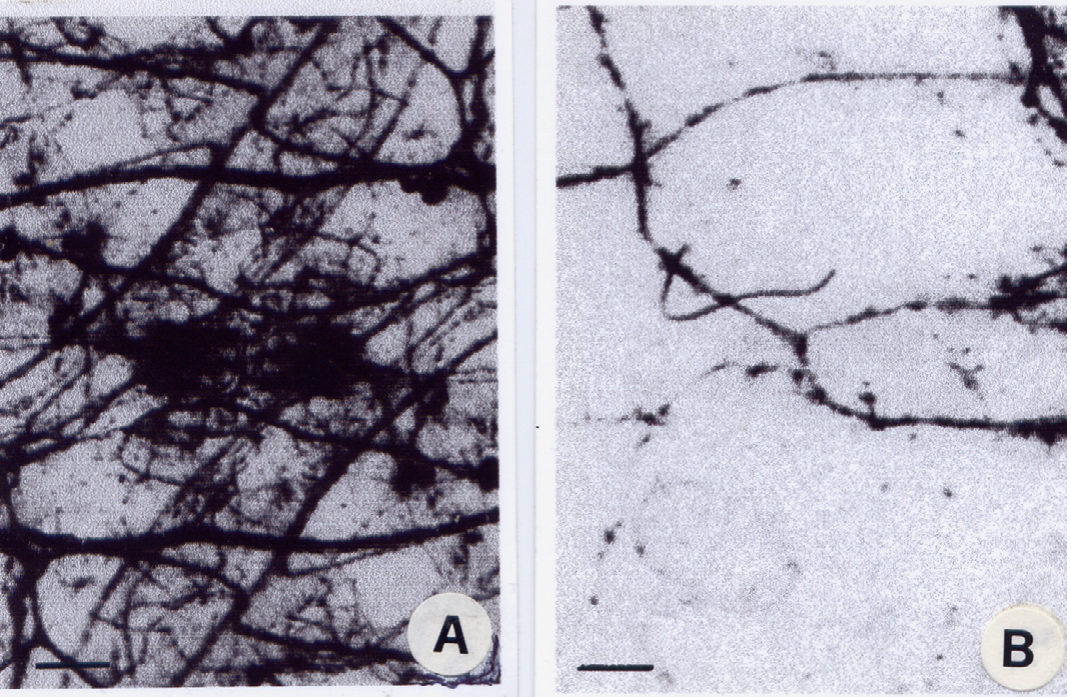
A and B. Light microscopy of total nerve fibers in a normal (A) and haemangiomatous (B) artery of the face stained using Bodian's method. The adventitia was separated from other layers of the artery wall and stretched on a slice. As can be seen, there are fewer nerve fibers in the haemangiomatous artery (B) than in the normal artery (A) (magnification 400×; bar 100 μm).

Bodian' s method stains all somatic and visceral nervous structures. Our results demonstrate that the nerve fibres in haemagiomatous vessels are poor and located in adventitial layer, while the medium layer and the endothelial layer are about devoid of nerve fibres (Fig. 2A and 2B).

**Figure 3:**
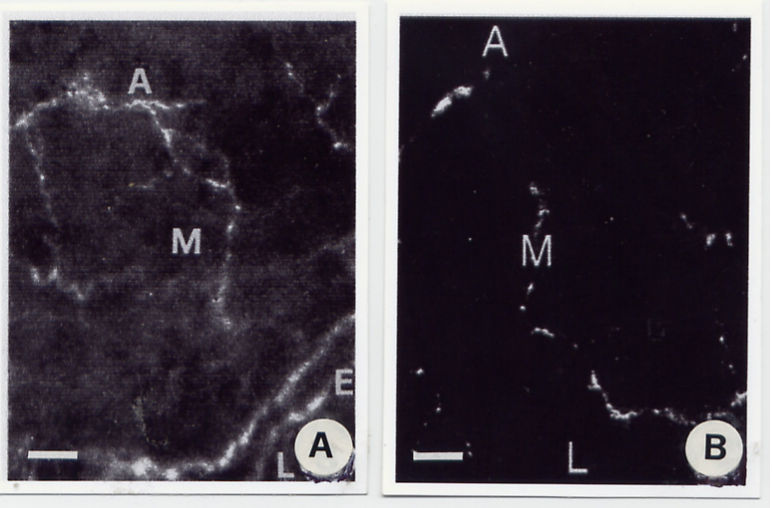
A and B. Fluorescent light microscopy of a transversal section of a normal (A) artery of the face stained using Falck's method for the staining of adrenergic nerve fibers compared with a haemangiomatous artery (B). A = adventitia; M = medium layer; E = endothelial layer; L = lumen (magnification 400×; bar 100 μm).

The haemangiomatous vessels were found to contain a number of ANF located in the adventitial layer. The other layer of the haemangiomatous vessels are about devoid of ANF. The total area of fluorescent structures is smaller, and the number of nervous varicosities, crossings and intersections of nerve fibres is lower in haemangiomatous vessels than in normal vessels (Fig. 3A and 3B; Table 2).

**Table 2:** Quantitative Analysis of images with statistical evaluation of the data of total, catecholaminergic, adrenergic nerve fibers (n. f.) and/or Beta-adrenergic receptors in the haemangiomatous and normal vessels.

QAI results	Haemangiomatous vessels	Normal vessels
Total n. f.	31.4 ± 2.8*	96.3 ± 3.1
Catecholaminergic n. f.	27.6 ± 3.3*	48.3 ± 2.8
Adrenergic n. f.	12.9 ± 2.8*	21.6 ± 3.2
Beta-adrenergic receptors	39.8 ± 3.2*	95.6 ± 3.6

All the results are expressed as C.U. (Conventional Units) ± SEM.

* P < 0.001 versus normal.

**Figure 4:**
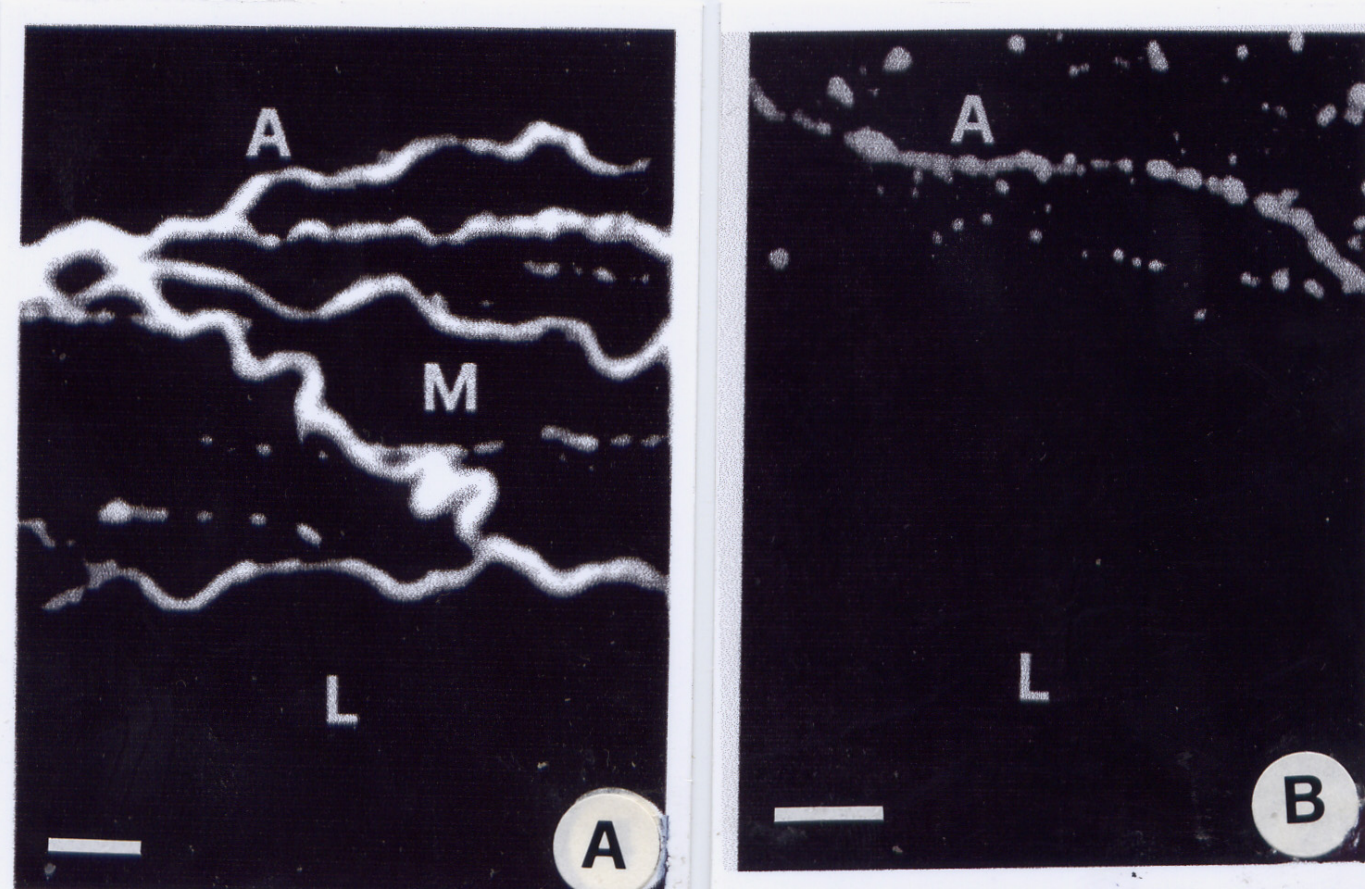
A and B. Fluorescent light microscopy of a transversal section of a normal (A) artery of the face stained using Qayyum's method, which stains the catecholaminergic nerve fibers. Results are compared with an analogue image from an haemangiomatous artery (B) A = adventitia; M = medium layer; L = lumen (magnification 400×; bar 100 μm).

Catecholaminergic nerve fibers were stained by means of Qayyum's method, using GIF induced fluorescence. Few nerve fibres in haemangiomatous vessels displayed GIF induced fluorescence. There was a lower number of catechola-minergic nerve fibres in haemangiomatous vessels than in normal vessels (Fig. 4A and 4B; Table 2).

**Figure 5:**
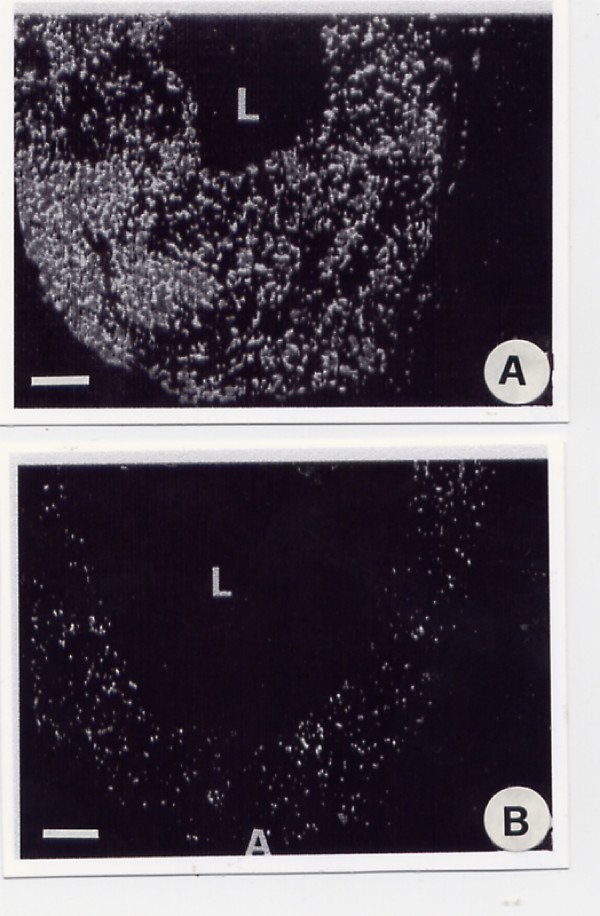
A and B. Fluorescent light microscopy of beta-adrenergic receptors contained in a transversal section of a normal (A) and an haemangioma-tous (B) artery of the face stained with fluorescent pindolole. The beta adrenergic receptors in the haemangiomatous arteries are located exclusively in the adventitia, while they lack in other layers. A = adventitia; L = lumen(magnification 800×; bar 100 μm).

Beta adrenergic receptors were stained by means of a fluoro-chrome linked to a beta adrenergic drug. Although beta-adrenergic receptors were found in both normal and haemangiomatous vessels, in the latter they were present in a decreased concentration and only in the adventitial layer (Fig. 5A and 5B; Table 2).

QAI yelded the morphometrical results listed in Table 2. As shown, the haemangiomatous vessels contained, in the whole wall, approximately 30% of the nerve fibres found in normal vessels. The majority of these nerve fibres were catecholaminergic and/or adrenergic, in nature. The beta-adrenergic receptors were strongly decreased in haemangiomatous vessels (about 30% than in normal vessels).

The statistical analysis showed that our results were significant, (<0.001, Table 2).

## Discussion

In normal blood-vessels nerve fibres are distributed in all the three layers of vascular wall (endothelium, smooth muscle medium layer, adventitia) in the hemangiomas the distribution of nerve fibres is lower than in normal conditions and nerve fibres are localized only in the adventitia, while the medium and the endothelial layers are about free of nerve fibres.

Moreover our results show that beta adrenergic receptors are evenly distributed in the wall of normal vessels, but unevenly distributed In the wall of the hemangiomatous vessels. There are fewer receptors than in normal tissues and located exclusively in the adventitia, while the medium and the endothelial layers are about free of beta- adrenergic receptors.

The research of specific markers [26] in rare head-facial syndromes can be helpful also for the identification of genes responsible of these diseases. Recently much progress has been made in the identification of genes responsible for rare head-facial syndromes. This has resulted in the ability to diagnose an increasing number of head-facial syndromes in the neonatal period by molecular genetic analysis. However, at the present time genetic testing for many of these conditions is only available from a small number of research laboratories. Transfer of genetic testing to diagnostic laboratories may make genetic testing more widely available but the costs of analysis are likely to be significant and may prevent accessibility to confirmatory genetic testing in many situations.

Newer, more rapid and, hopefully, cheaper techniques of mutation analysis may make accessibility to genetic testing more widely available.

Our results on the sympathetic nerve fibres of the haemangiomatous vessels can be useful for the studies of specific sympathetic markers and their specific genes.

## Competing interests

The author(s) declare that they have no competing interests.

## Authors' contributions

G I performed the surgical techniques and produced the biopsies used in this study. AT performed all clinical analysis and digital x-ray image acquisition. SC described and performed the laser therapy, set up the digital x ray acquisition as well as all the clinical analysis. CC conceived the study and its design, produced all experimental results, performed the statistical analyses and wrote the manuscript drafts. All authors read and approved the final manuscript.
